# Association left ventricular lead and ventricular arrhythmias after upgrade to cardiac resynchronization therapy in patients with implantable cardioverter defibrillators

**DOI:** 10.1002/clc.23192

**Published:** 2019-05-13

**Authors:** Mitsuharu Kawamura, Shuhei Arai, Kosuke Yoshikawa, Toshihiko Gokan, Ko Ogawa, Akinori Ochi, Yoshimi Onishi, Yumi Munetsugu, Hiroyuki Ito, Tatsuya Onuki, Youichi Kobayashi, Toshiro Shinke

**Affiliations:** ^1^ Division of Cardiology, Department of Medicine Showa University School of Medicine Tokyo Japan

**Keywords:** cardiac resynchronization therapy, heart failure, lead threshold, upgrade, ventricular tachycardia

## Abstract

**Background:**

There are some controversial reports related to the pro‐arrhythmic or anti‐arrhythmic potential of cardiac resynchronization therapy (CRT) and little is known about the relationship between ventricular arrhythmia (VA) and left ventricular (LV)‐lead threshold.

**Hypothesis:**

Upgrade CRT is anti‐arrhythmic effect of VA with implantable cardioverter‐defibrillator (ICD) patients and has a relationship with the incident of VA and LV‐lead threshold.

**Methods:**

Among 384 patients with the implantation of CRT‐defibrillator (CRT‐D), 102 patients underwent an upgrade from ICD to CRT‐D. We divided patients into three groups; anti‐arrhythmic effect after upgrade (n = 22), pro‐arrhythmic effect (n = 14), and unchanging‐VA events (n = 66). The VA event was determined by device reports. We described the electrocardiography parameters, LV‐lead characteristics, and clinical outcomes.

**Results:**

Before upgrade, the numbers of VA were 305 episodes and the numbers of ICD therapy were 157 episodes. While after upgrade, the numbers of VA were 193 episodes and the number of ICD therapy were 74 episodes. Ventricular tachycardia cycle length (VT‐CL) after upgrade was significantly slower as compared to those with before upgrade. Pro‐arrhythmic group was significantly higher with delta LV‐lead threshold (after 1 month—baseline) as compared to those with anti‐arrhythmic group (0.74 vs −0.21 V). Furthermore, pro‐arrhythmic group was significantly bigger with delta VT‐CL (after 3 months—before 3 months) as compared to those with anti‐arrhythmic group (*P* = .03).

**Conclusions:**

We described upgrade‐CRT was associated with reduction of VA, ICD therapies and VT‐CL. While 14 patients had a pro‐arrhythmic effect and LV lead threshold might be associated with VA‐incidents.

## 1 INTRODUCTION

Cardiac resynchronization therapy with defibrillator (CRT‐D) is an approved treatment for patients with advanced staged of heart failure in patients with wide QRS, and this therapy is associated with reduction in symptoms, improvement in left ventricular ejection fraction (LVEF), and decrease in hospitalization, and mortality.^1^, ^2^, ^3^ However, there is a controversy regarding the effect of CRT‐D on the risk of ventricular arrhythmia (VA). Recent studies reported that there were no significant difference in total VA between CRT‐D patients and implantable cardioverter‐defibrillator (ICD) patients.^4^, ^5^ Some studies reported a reduction in the risk of VA associated with CRT and suggested that the significant improvements in LV volumes could account for this effect.^6^, ^7^ The other studies reported that LV epicardial activation in CRT might cause dispersion of repolarization and prolongation of the QT interval, therefore, VA was increased after CRT‐D therapy.^8^, ^9^, ^10^ Theis et al^11^ reported that increased LV stimulus was associated with longer QT interval. There are some controversial reports related to the pro‐arrhythmic or anti‐arrhythmic potential of CRT therapy and it seems necessary to further investigate the pro‐arrhythmic potential and cause of CRT. We investigated whether upgrade of CRT‐D is anti‐arrhythmic effect of VA with ICD patients and we evaluated the association between LV lead threshold and incidents of VA.

## 2 METHODS

### 2.1 Patients and study protocol

Among 384 patients with the implantation of CRT‐D, 102 patients (27%) underwent an upgrade from ICD to CRT‐D between September 2006 and July 2016. The follow‐up period of VA event was 1 year before upgrade and 1 year after upgrade CRT‐D. The follow‐up period of mortality and hospitalization of cardiac event was 2 years after upgrade CRT‐D. Patients with the percent of bi‐ventricular pacing were under 50% (range 0%‐50%) after upgrade CRT‐D and withdrawal during follow‐up period were excluded. The anti‐arrhythmic effect of CRT‐D was defined as 80% reduction of VA episodes by device reports after upgrade. The pro‐arrhythmic effect was defined as 80% increase of VA episodes by device reports after CRT‐D upgrade. The other patients were defined as unchanging VA group. Before upgrade, all patients with advanced heart failure were New York Heart Association (NYHA) functional classes II, III or IV, and decreased LVEF (40% or less) and wide QRS complex (> 120 mseconds).

Patients were classified as responders if their LVEF increased by at least 20%, and/or the LV‐ESV decreased by at least 15% with respect to baseline (variations were considered as relative values). Patients were defined non‐responders if they did not reach both the above pre‐specified echocardiographic changes. All patients gave written informed consent before catheter ablation. This study was approved by the Institutional Committee at our institution.

### 2.2 Measurements

This study evaluated VA episodes before 3, 6, and 12 months for CRT‐D upgrade and after 3, 6, and 12 months for CRT‐D upgrade by the device reports. In addition, we measured ICD therapies by the device reports, including shock therapy and anti‐tachycardia pacing (ATP) therapy. The QRS duration, QT interval, and QTc by standard 12 lead electrocardiograms (ECG) were measured before CRT upgrade and after 6 months follow‐up. QT intervals were measured from a beginning of the QRS complex to the end of the T wave, which was defined as return to baseline in each ECG lead. If U waves were present, QT interval was measured to the nadir of the curve between the T and U waves. Corrected QT was calculated from the values in second using Bazzett equation (QTc = QT/√R−R). The LV lead pacing was performed at decrementing stimulus amplitudes from the maximum allowable output of the device to LV capture threshold immediately after CRT upgrade and 3 months later. Echocardiography was performed before upgrade and after 3 months. The LVEF was assessed by biplane Simpton's equation using the apical 4‐ and 2‐chamber views. LV mass was estimated by using the corrected American Society of Echocardiography (ASE) method: 0.8 × (1.04 × ([IVSd + LVIDd + PWTd] ‐ LVIDd)) + 0.6 and normalized to body surface area.^12^, ^13^

Scar area was defined as ischemic position by coronary angiography and by echocardiography. Blood sample was measured before upgrade and 3 months later.

### 2.3 CRT‐D implantation and definitions

We performed LV pacing lead into a branch of the coronary sinus (n = 102). The LV lead was implanted transvenously through the coronary sinus tributaries and placed preferably to stimulate the lateral or postero‐lateral LV wall. Ventricular tachycardia (VT) zone was defined as the ventricular rate up to 150 beats/min and fast VT was defined as the ventricular rate up to 188 beats/min. And, ventricular fibrillation (VF) zone was defined as the ventricular rate up to 250 beats/min. The ICD defibrillators were programmed as follows: VT monitor zone was programmed in all patients (150‐188 beats/minutes). Any VT faster than 188 beats/minutes was attempted to be terminated with anti‐tachycardia pacing or device shocks. Any VF faster than 250 beats/minutes was directly attempted to be terminated by device shocks. The anti‐tachycardia pacing was attempted with eight pulses at 88% of the measured cycle length (CL) with a 10‐ms decrement between bursts. The initial device shock was attempted at the defibrillation threshold plus at least 10 J. The remaining device shock should be maximal energy shocks. The ICD and CRT‐D therapies were programmed according to the attending physician's discretion.

### 2.4 Statistical analysis

Data are presented as mean ± SD. Multiple‐group comparisons were obtained by ANOVA. Categorical data are summarized as frequencies and percentages. Differences in baseline characteristics among patients with unchanging VA, pro‐arrhythmic group, and anti‐arrhythmic group were analyzed using unpaired Student *t* tests. The paired Student *t* test was used to compare continuous data within the subgroups during follow‐up. The hazard ratio and its confidence intervals were estimated using the Cox regression model. *P*‐values <.05 were considered statistically significant. The authors had full access to and take full responsibility for the integrity of the data. All authors have read and agree to the manuscript as written.

## 3 RESULTS

### 3.1 Patient characteristics

We investigated and analyzed a total of 102 patients which received upgrade from ICD to CRT‐D. There were 83 men and 19 women, and their mean age was 60 ± 14 years. The mean LVEF before upgrade CRT‐D was 27 ± 12%. Ischemic heart disease was recorded 52 (51%) patients. Before and after CRT‐D upgrade, NYHA function class, ECG finding, LVEF, and creatinine were summarized in Table 1. QRS duration with post‐upgrade was significantly narrower as compared to those with pre‐upgrade. QT interval with post‐upgrade was significantly longer as compared to those with pre‐upgrade. Upgrade to CRT‐D resulted in a significant improvement of LVEF and LV mass index.

**Table 1:** Change in characteristics before and after CRT‐D upgrade

	Before upgrade	After upgrade	*P*‐value
NYHA functional class			
I	0 (0%)	1 (1%)	.31
II	27 (26%)	44 (42%)	.06
III	65 (64%)	53 (51%)	.12
IV	10 (10%)	4 (3%)	.08
Electrocardiography			
QRS duration (msec)	161 ± 48	146 ± 41	<.001
QT interval (msec)	446 ± 81	459 ± 86	.04
Corrected QT interval	487 ± 83	496 ± 88	.07
VT‐CL (bpm)	203 ± 56	181 ± 48	.001
Echocardiographic data			
LVEF (%)	27 ± 13	36 ± 15	<.001
LV mass index (g/m^2^)	124 ± 32	110 ± 28	<.001
Biomarker			
Creatinine (mg/dL)	1.29 ± 0.47	1.36 ± 0.48	.36

Abbreviations: LV, left ventricular; LVEF, left ventricular ejection fraction; NYHA, New York Heart Association; VT‐CL, ventricular tachycardia cycle length.

### 3.2 Number of VA episodes and ICD therapies

**Figure 1:**
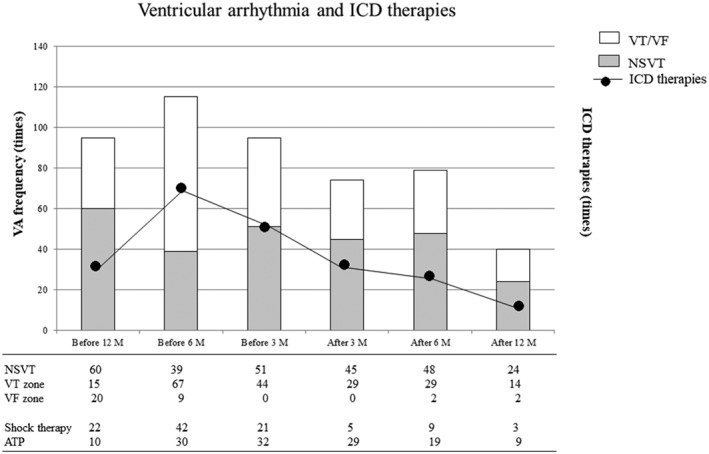
A bat graph shows the number of ventricular arrhythmia before and after CRT‐D upgrade. A line graph shows the number of ICD therapies before and after CRT‐D upgrade. ATP, anti‐tachycardia pacing; CV, cardioversion; CRT‐D, cardiac resynchronization therapy‐defibrillator; ICD, implantable cardioverter‐defibrillator; NSVT, non‐sustained ventricular tachycardia

Figure 1 showed the number of VA episodes and ICD therapies before and after upgrade. One patient was excluded this figure due to the electrical storm after upgrade and received many shock therapies. Before upgrade, the numbers of VA were 305 episodes (NSVT; 150 events, VT zone; 126 events, and VF zone; 29 events) and the numbers of ICD therapy were 157 episodes (shock therapy; 85 events and ATP therapy; 72 events). While after upgrade, the numbers of VA were 193 episodes (NSVT; 117 events, VT zone; 72 events and VF zone; 4 events) and the numbers of ICD therapy were 74 episodes (shock therapy; 17 events and ATP therapy; 57 events). Upgrade CRT‐D had 43% reduction with VT zone events and 86% reduction with VF zone events. Furthermore, upgrade CRT‐D had 80% reduction with shock therapy.

### 3.3 Comparison of baseline characteristics and LV lead findings among three groups

**Figure 2:**
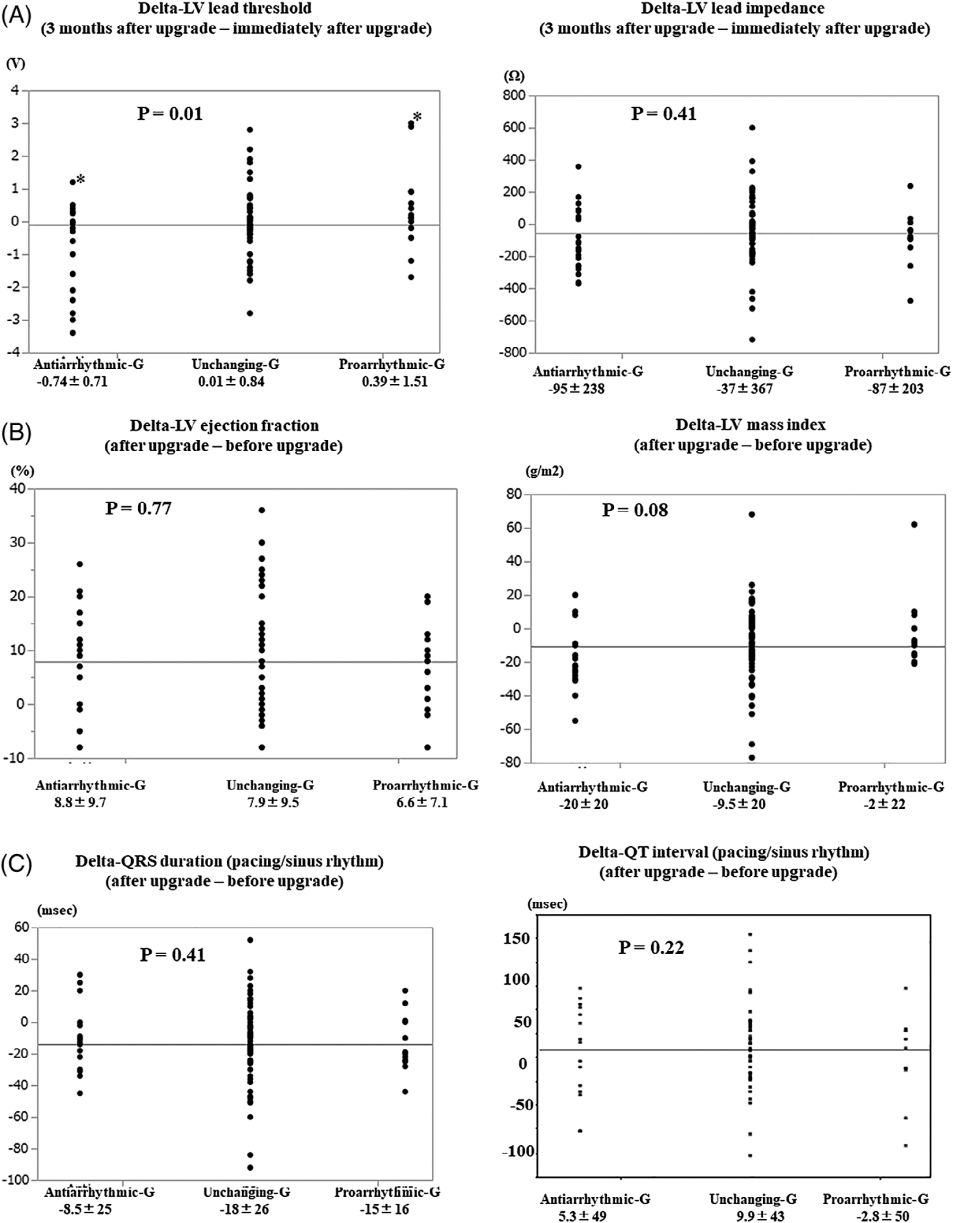
A, The change in LV lead findings between 3 months after upgrade and immediately after upgrade. B and C, Cardiac echo findings and ECG findings between before CRT‐D upgrade and after CRT‐D upgrade. CRT‐D, cardiac resynchronization therapy‐defibrillator; ECG, electrocardiograms; EF, ejection fraction; LV, left ventricular; VT, ventricular tachycardia

We divided into three groups; unchanging VA group (n = 66), anti‐arrhythmic effect group (n = 22), and pro‐arrhythmic effect group (n = 14). Table 2 showed the characteristics among three groups. There were no significant differences except delta VT‐CL. Delta VT‐CL in pro‐arrhythmic group was significantly bigger as compared to those with anti‐arrhythmic group (21 ± 12 vs −25 ± 15 bpm, *P* = .03). Delta QRS duration during VT in pro‐arrhythmic group tended to be bigger as compared to those with anti‐arrhythmic group (21 ± 14 mseconds vs −15 ± 10 bpm, *P* = .08). Figure 2A showed that the delta‐LV lead threshold (LV threshold 3 months after upgrade ‐ LV threshold immediately after upgrade) in pro‐arrhythmic group was worse, while anti‐arrhythmic group was better with delta‐LV lead threshold. Figure 2B showed that the delta‐LVEF was not significantly differenced among three groups, while the delta‐LV mass index with anti‐arrhythmic group tended to be more improved as compared to those with pro‐arrhythmic group. Figure 2C showed that the delta‐QRS duration and delta‐QT interval were not significantly difference among three groups. Table 3 showed an individual characteristics and data of pro‐arrhythmic group. Eleven patients (79%) improved LVEF and 12 patients (86%) improved QRS duration after upgrade. One patient had decreased VT‐CL and six patients had increased VT‐CL after upgrade. Seven patients had no VT/VF events before upgrade. Nine patients had worsened LV threshold after 1 month and 11 patients were positioned LV lead on the posterolateral wall and three patients were positioned on the anterolateral wall.

**Table 2:** Comparison of characteristics among three groups

	Unchanging (n = 66)	Pro‐arrhythmic (n = 14)	Anti‐arrhythmic (n = 22)	*P*‐value
Age (years)	60 ± 16	61 ± 9	60 ± 13	.97
Sex (male)	53 (81%)	11 (78%)	19 (87%)	.59
BMI (kg/m^2^)	25 ± 4	28 ± 6	26 ± 5	.36
Secondary prevention	31 (47%)	11 (78%)	12 (53%)	.08
Ischemic heart disease	34 (51%)	8 (57%)	10 (45%)	.45
Non‐ischemic heart disease	32 (49%)	6 (43%)	12 (55%)	.55
History of AF	29 (44%)	7 (50%)	9 (40%)	.42
Inappropriate therapy	2 (3%)	1 (7%)	1 (5%)	.63
QRS duration (msec)	161 ± 38	161 ± 23	159 ± 20	.98
QT interval (msec)	444 ± 67	466 ± 40	442 ± 40	.57
QTc	479 ± 61	490 ± 25	511 ± 49	.16
LV ejection fraction (%)	27 ± 6	28 ± 9	25 ± 8	.12
LV mass index (g/m^2^)	124 ± 31	112 ± 16	131 ± 21	.26
CRT responder	49 (74%)	10 (71%)	18 (81%)	.48
Biventricular pacing rate	88%	92%	89%	.58
LV lead position				
Posterior/post‐late	53 (81%)	11 (78%)	20 (93%)	.27
Anterior/antero‐late	13 (19%)	3 (22%)	1 (7%)	.27
LV threshold (V)	1.18 ± 0.86	0.95 ± 1.1	1.45 ± 0.61	.36
Medication				
ACE‐I/ARB	43 (65%)	9 (65%)	13 (59%)	.55
Beta‐blockers	49 (74%)	11 (78%)	18 (81%)	.65
Amiodarone/Sotalol	18 (27%)	5 (35%)	7 (32%)	.48
Digoxin	10 (15%)	2 (14%)	4 (18%)	.42
Statin	27 (41%)	7 (50%)	11 (50%)	.45
Deterioration of NYHA	4 (6%)	0 (0%)	2 (9%)	.42
Creatinine (mg/dL)	1.31 ± 0.83	1.41 ± 1.38	1.17 ± 0.42	.76
Delta VT‐CL (bpm)	−14 ± 8	21 ± 12	−25 ± 15	.03
Delta QRS duration (VT, msec)	−12 ± 9	21 ± 14	−15 ± 10	.08

Abbreviations: ACE‐I, angiotensin converting‐enzyme inhibitors; AF, atrial fibrillation; ARBs = angiotensin receptor blockers; BMI, body mass index; CRT, cardiac resynchronization therapy; EF, ejection fraction; LV, left ventricular; NYHA = New York Heart Association; VT, ventricular tachycardia; VT‐CL, ventricular tachycardia cycle length.

**Table 3:** Patients with the pro‐arrhythmic effect of ventricular arrhythmias after upgrade

Case	1	2	3	4	5*	6	7	8	9	10	11	12	13	14
Age (year)	60	68	48	81	61	59	55	72	56	65	72	53	55	63
Sex	F	M	M	M	F	M	M	M	F	M	M	M	M	M
LVEF														
Before (%)	31	30	30	25	31	30	29	24	32	27	30	30	25	32
After (%)	35	40	36	42	30	45	33	40	40	35	28	35	42	45
QRS‐d (pacing)														
Before (ms)	160	165	170	178	162	163	180	145	140	170	142	160	166	152
After (ms)	140	143	160	150	132	135	180	135	130	158	130	138	168	145
QRS‐d (VT)														
Before (ms)	180	210	190	(−)	(−)	160	210	(−)	(−)	(−)	(−)	180	160	(−)
After (ms)	230	210	220	200	190	170	200	175	180	158	130	200	200	145
VT‐CL														
Before (bpm)	180	170	200	(−)	(−)	190	210	(−)	(−)	(−)	(−)	170	180	(−)
After (bpm)	200	210	220	170	196	200	190	184	178	200	168	200	210	180
Lead threshold														
Before (V)	0.8	0.7	1.2	1.5	1.2	0.8	1.2	0.8	0.5	0.8	0.6	1.2	1.5	0.5
After (V)	2.2	1.2	1.2	2.3	0.8	1.5	1.2	1.5	1.2	0.5	1.2	1.5	1.2	1.2
LV lead site	P‐L	P‐L	A‐L	P‐L	P‐L	A‐L	P‐L	P‐L	P‐L	A‐L	P‐L	P‐L	P‐L	P‐L

Abbreviations: A‐L, anterolateral, LVEF, left ventricular ejection fraction, P‐L, posterolateral, QRS‐d, QRS duration, VT‐CL, ventricular tachycardia cycle length.

5* This patient had an electrical storm after upgrade CRT‐D.

### 3.4 Relation between lead position and scar area in ischemic cardiomyopathy

In ischemic cardiomyopathy, 34 patients were in unchanging VA group, eight patients were in pro‐arrhythmic group and 10 patients were in anti‐arrhythmic group (Table 2). In pro‐arrhythmic group, five patients had scar areas at posterior and anterior area, two patients had scar areas at posterior and one patient had scar area at anterior. Furthermore, six patients were positioned with LV lead at posterior lateral and one patient was positioned with LV lead at anterior lateral. Six patients (6/8, 75%) had a same area with LV lead position and a scar area in pro‐arrhythmic group. In anti‐arrhythmic group, four patients had scar areas at posterior and anterior area, two patients had scar areas at posterior and four patients had scar area at anterior. Furthermore, eight patients were positioned with LV lead at posterior lateral and two patients were positioned with LV lead at anterior lateral. Five patients (4/10, 40%) had a same area with LV lead position and a scar area in anti‐arrhythmic group.

## 4 DISCUSSION

### 4.1 Main findings

The most important finding of this study is that upgrade to CRT‐D decreases the frequent of VA episodes, ICD therapies, and VT‐CL. While 14 patients had more frequently with VA episodes after upgrade. We described a direct relationship between the frequent of VA and LV lead threshold in patients with upgrade from ICD to CRT‐D. In the pro‐arrhythmic group, LV lead threshold after 3 months was significantly worse as compared to those with immediately after upgrade, while LV lead threshold in anti‐arrhythmic group after 3 months significantly improved as compared to those with immediately after upgrade. Delta VT‐CL in pro‐arrhythmic group was significantly bigger as compared to those with anti‐arrhythmic group.

### 4.2 Anti‐arrhythmic and pro‐arrhythmic effect of CRT therapy

Several studies demonstrated the upgrade from ICD to CRT‐D was associated with a reduction of VA events and ICD therapies.^14^, ^15^ These results were similar to our study, while these studies included a small number of patients. During permanent CRT‐D therapies, reduction of a ventricular conduction delay, leading to a decrease in the occurrence of reentry, avoidance of pause‐dependent tachyarrhythmia and reduction in the circulating levels of norepinephrine, all known mechanisms that might trigger VA.^5^, ^16^ Other studies described that the reduction of VA in patients with CRT might be explained by the CRT‐induced reverse remodeling.^5^, ^17^, ^18^, ^19^ Furthermore, in the MADIT‐CRT (Multicenter Automatic Defibrillator Implantation Trial‐Cardiac Resynchronization Therapy), the extent of reverse ventricular remodeling during the first year after device implantation was inversely related to the risk of future VA events. And, every 10% reduction in LV end‐systolic volume was associated with a significant reduction in all the following endpoint: VA, VA/death, and VF events. These results were also observed for other measures of reverse remodeling including reduction in LV end‐systolic volume, LV mass, and increase in LVEF.^20^ We described that LV mass index in an anti‐arrhythmic group tended to improve as compared to those with pro‐arrhythmic group. These results suppose that the reverse remodeling induced by CRT results in both mechanical and electrical stability of the LV, leading to reduce VA events. Furthermore, these results might reduce VA‐CL after upgrade CRT. Several studies have suggested that CRT (epicardial LV pacing) may promote VA, possibly due to reversal of the normal sequence of activation induced by LV epicardial pacing that may lead to prolongation of the QT interval and an increase in the transmural dispersion of repolarization.^8^, ^9^ Gasparini et al reported that electrical storm occurred in 45 (7%) of 631 CRT‐D patients.^21^ Our study was similar to indicate that upgrade CRT‐D significantly decreased VA episodes, while 14 patients increased VA episodes after upgrade CRT‐D. Therefore, it might be continuing controversial discussion related to pro‐arrhythmic or anti‐arrhythmic potential of CRT.

### 4.3 Relationship between the frequent of VA and LV lead threshold and location

This study demonstrated a relationship between the frequent of VA and LV lead threshold in patients with upgrade from ICD to CRT‐D. Pro‐arrhythmic group significantly increased LV lead threshold after 3 months of upgrade, however, anti‐arrhythmic group improved it. This is the first study to examine the relationship between the frequent of VA and LV lead threshold in patients with upgrade of CRT. The mechanism was not clear yet, however, support report indicated that increase LV stimulus was associated with faster trans‐ventricular conduction time, changes in myocardial depolarization, and longer QT intervals. Increased stimulus intensity led to a prolongation of QTc from 539 ± 45 (0.5 V > LV threshold) to 559 ± 46 mseconds (5 V < LV threshold). These findings had important implications on the relationship of programmed LV pacing output to pacing‐induced proarrhythmia.^11^ Furthermore, high epicardial pacing output might induce the bigger delta VT‐CL and delta QRS duration during VT. However, we will need to clear the mechanism of the bigger delta VT‐CL and delta QRS duration during VT in pro‐arrhythmic group. The reason to increase LV lead threshold was mainly LV lead dislodgement with loss capture, however, LV lead threshold sometimes increased without obvious lead dislodgement. We used the traditional bipolar lead and we only chose four different LV pacing configurations. Recent study demonstrated that a quadripolar CS lead has been designed to provide 10 options for LV pacing and it is useful for dislodgement and phrenic nerve stimulation.^21^ LV quadripolar lead might be useful for VA reduction for CRT patients, too.^22^ In the MADIT‐CRT trial,^20^ posterior or lateral LV lead location was associated with decreased risk of arrhythmic events in comparison with anterior lead location. Another report suggested that there was no significant difference in the susceptibility to arrhythmic events regarding LV lead positioning.^23^ In our study, there was no significant relationship between LV lead position and VA events, too. However, further studies will be needed to determine the relationship between LV lead characteristics and VA events.

### 4.4 Relationship between the frequent of VA and clinical outcome

Thijssen et al^24^ reported that after upgrade from ICD to CRT‐D, non‐responders to CRT showed a significant increase in VA burden requiring appropriate device therapy. And the other study reported that within the initial 6‐month post‐CRT therapy, 20% of patients received an appropriate ICD therapy. Patients improving on NYHA class have less VT/VF episodes than non‐responders.^25^ In our study, 14 patients increased the frequent of VA. However, CRT non‐responder is only two patients. Furthermore, there was no significant difference with the rate of non‐responder to CRT among three groups. Therefore, there was no relationship between the frequent of VA and CRT responder, NYHA class in our study. Anti‐arrhythmic drugs and beta‐blockers reduced VA event and VA events might be affected by these drugs. However, there was no significant difference with anti‐arrhythmic drugs and beta‐blockers among three groups.

### 4.5 Study limitations

The study has several limitations. First, the number of subjects was relatively small. However, we believe that this study is an adequate evaluation as there was a significant difference between increase VA group and decrease VA group. Second, this study was a retrospective observational analysis of prospectively assessed data evaluating the frequent of VA before and after CRT‐D upgrade. However, the frequent of VA was investigated by the device reports. Therefore, the data was full confidence. However, further studies are needed to clarify the relationship between the frequent of VA and LV lead characteristics.

## 5 CONCLUSIONS

This is the first study to examine the relationship between the frequent of VA and LV lead threshold in patients with upgrade from ICD to CRT‐D. Furthermore, this study demonstrates that upgrade therapy reduces the frequent of VA, ICD therapies.
